# Supplementary material for: Very-Low-Frequency transmitters bifurcate energetic electron belt in near-earth space
Source: Nat Commun. 2020 Sep 24;11:4847. doi: 10.1038/s41467-020-18545-y (PMC7518438; doi:10.1038/s41467-020-18545-y)
Supplement: Supplementary file 1 — Supplementary Information [file 41467_2020_18545_MOESM1_ESM.pdf]

Supplementary Information for

Very-Low-Frequency Transmitters Bifurcate Energetic Electron Belt in Near-Earth Space

Man Hua[1, 2], Wen Li[2*], Binbin Ni[1,3*], Qianli Ma[2,4], Alex Green[2], Xiaochen Shen[2], Seth G. Claudepierre[4,5], Jacob Bortnik[4], Xudong Gu[1], Song Fu[1], Zheng Xiang[1], and Geoffrey D. Reeves[6,7]

[1] Department of Space Physics, School of Electronic Information, Wuhan University, Wuhan, Hubei, China.

[2] Center for Space Physics, Boston University, Boston, Massachusetts, USA.

[3] CAS Center for Excellence in Comparative Planetology, Anhui, Hefei, China.

[4] Department of Atmospheric and Oceanic Sciences, University of California, Los Angeles, California, USA.

[5] Space Sciences Department, The Aerospace Corporation, EI Segundo, CA, USA.

[6] Space Science and Applications Group, Los Alamos National Laboratory, Los Alamos, NM, USA.

[7] Space Sciences Division, New Mexico Consortium, Los Alamos, NM, USA.

***Correspondence authors**. Emails: wenli77@bu.edu; bbni@whu.edu.cn

Supplementary Methods

RBSPICE and MagEIS instruments onboard the Van Allen Probes. Launched in August 2012, the twin Van Allen Probes operated in nearly the same highly elliptical, low inclination orbits with the perigees of ~1.1 Earth radii (R_E) and the apogees of ~5.8 R_E[1]. The identical instrument set of particle measurements onboard both probes, including Radiation Belt Storm Probes Ion Composition Experiment (RBSPICE)[2], Magnetic Ion Spectrometer (MagEIS)[3,4] and Relativistic Electron Proton Telescope (REPT)[4,5], provide high quality measurements to study the dynamic variations of radiation belt electrons from tens of keV to ~20 MeV.

In the present study, we used data acquired by RBSPICE to investigate the temporal evolution of energetic electrons at $L < 3$ in the Earth's inner magnetosphere. The RBSPICE instrument measures electrons at energies of ~ 25 keV–1 MeV with fine resolution (*i.e.,* 38 energy channels in the energy range of ~ 25–200 keV[2]), which is suitable to analyze the details of radiation belt electron energy spectra and their spatiotemporal variations (Fig. 1c-h).

At the same time, the MagEIS instrument measures electrons at energies of ~ 30 keV – 4 MeV with 8 energy channels below 200 keV[3]. Albeit with fewer energy channels, MagEIS observed the bifurcated structure of electron fluxes similar to RBSPICE. Electron flux measurements from MagEIS onboard both Van Allen Probes are shown in Supplementary Fig. 1, in the same format as Fig. 1, showing a gradual decay below 100 keV in the radial profile of electron flux at L ~ 2.0–2.5 during the period from 20 February to 6 March in 2016. Meanwhile, the local flux minima at L ~ 2.0–2.2 became pronounced with time, leading to the bifurcated electron belt (indicated by the arrows in Supplementary Fig. 1c-e). Despite fewer energy channels, the energies of radial electron flux minima are approximately consistent with the minimum first-order cyclotron resonant energies of electrons (white dashed curves in

Supplementary Fig. 1f-h) interacting with in situ observed VLF transmitter signals at 24 kHz. Identification of the near-Earth bifurcated energetic electron belt using both RBSPICE and MagEIS measurements unambiguously confirms its formation, and justifies our use of the RBSPICE dataset with finer energy resolution for further quantitative analyses.

High Frequency Receiver (HFR) onboard the Van Allen Probes. The Electric and Magnetic Field Instrument Suite and Integrated Science (EMFISIS)[6] onboard the Van Allen Probes provides high quality measurements of wave spectral intensity spanning the frequencies from 10 Hz to 400 kHz. As a part of the EMFISIS instrument, the high-frequency receiver (HFR) provides one component of the wave electric field in 82 logarithmically spaced frequency bins over the frequency range of 10–400 kHz in the plane perpendicular to the spin axis roughly directed toward the Sun[6].

With the operating frequencies at 10–30 kHz, the signals of VLF transmitters that propagate through the ionosphere can be detected by HFR if their wave power in space is above the instrument noise level. As shown in the observation of VLF transmitter waves on 23 February (Fig. 2a), signatures of VLF transmitter waves were clearly captured by the HFR measurements with the dominant wave power in the frequency range of ~18–26 kHz. In general, VLF transmitter waves observed at $L < 1.7$ at the frequency of ~20 kHz propagate mostly unducted[7], mainly corresponding to the powerful 19.8 kHz NWC transmitter located at North West Cape in Australia ($L \sim 1.4$) and also the 21.4 kHz NPM transmitter in Hawaii ($L \sim 1.2$). Another group of VLF transmitter waves observed at $L > 1.7$ at higher frequencies of ~24 kHz is mainly ducted[8], primarily corresponding to the 23.4 kHz DHO38 transmitter ($L \sim 2.4$) located in Germany, and 24 kHz NAA transmitter ($L \sim 2.7$) and the 24.8 kHz NLK transmitter ($L \sim 2.9$) located in North America.

Furthermore, the HFR instrument provides burst measurements with finer time resolution, which show that narrowband VLF transmitter waves can be frequently observed. Supplementary Fig. 3 provides an example of the observed VLF transmitter waves from the survey-mode (Supplementary Fig. 3a) and burst-mode (Supplementary Fig. 3b) HFR measurements by Van Allen Probe A during 02:57 – 03:02 UT on 22 February 2016. The VLF transmitter waves were observed on the nightside over *L*-shells of 2.2 – 2.4 near the geomagnetic equator. The wave electric power peaked at ~18 kHz and 24 kHz, exhibiting a strong coherent structure.

Models of VLF transmitter waves and natural plasma waves based on observations. Apart from VLF transmitter signals, there are naturally occurring magnetospheric plasma waves which also play a significant role in wave-particle interaction processes to alter the electron dynamics at low *L*-shells, including lightning-generated whistlers (LGWs), plasmaspheric hiss, and magnetosonic waves. It is believed that the long-term energetic electron loss is largely controlled by pitch-angle scattering driven by whistler mode waves[9-11]. Plasmaspheric hiss is generally regarded as a broadband whistler mode emission from tens of Hz to several kHz, peaking at a few hundred Hz, inside the plasmasphere or plasmaspheric plume[12-18]. Hiss waves drive electron pitch-angle diffusion on timescales from a few days to hundreds of days with a strong energy dependence[9,10,18-24], which is dominantly responsible for the formation of the slot region[25] and the reversed electron energy spectrum[26,27]. A recent study also suggested that strong hiss waves with amplitudes up to a few hundred pT can be observed over $L = 2 – 3$ on the dayside with the elliptical polarization and oblique propagation[28-30], which can potentially drive the sporadic electron precipitation[30]. Unducted LGWs can form a population of magnetospherically reflected whistlers trapped in the inner magnetosphere[31] and contribute to the loss of radiation belt electrons[9,21,32]. As a linearly polarized wave mode propagating almost perpendicularly to the

geomagnetic field, magnetosonic waves primarily occur within ~3° of the geomagnetic equator both inside and outside the plasmasphere, commonly in the *L*-shell range of ~2–7 with frequencies from the proton cyclotron frequency to the lower hybrid resonant frequency[33-37]. Magnetosonic waves are found to be capable of accelerating radiation belt electrons[38] and producing electron butterfly pitch-angle distributions via Landau resonance[33,39-42].

To comprehensively understand the electron dynamics observed at L ~ 1.5–3.0 during the geomagnetically quiet time (Fig. 1), all the plasma waves that can occur at these *L*-shells need to be considered. Due to a lack of full MLT coverage during the 15-day period using the Van Allen Probes measurements, we adopt both statistical information and in situ observations of wave parameters for the four wave modes. For VLF transmitter waves, we adopt a statistical wave frequency spectrum model, based on a survey of the Van Allen Probes EMFISIS HFR measurements during the period from 2012 to 2016, with the lower and upper cutoff frequencies at 10 kHz and 30 kHz, respectively, the frequency bandwidth of 1 kHz, and the peak wave frequency of 20 kHz (24 kHz) below (above) L = 1.7[43]. We adopt a statistical wave frequency spectrum model of LGWs with the lower and upper cutoff frequencies at ~100 Hz and ~10 kHz[44], respectively, based on the Van Allen Probes EMFISIS waveform data over the entire Van Allen Probes era. The statistical model of hiss wave frequency spectrum during modestly disturbed periods[12], based on the Van Allen probes EMFISIS survey mode data, is adopted with the lower and upper cutoff frequencies at ~20 Hz and ~4 kHz, respectively. The adopted frequency spectrum of magnetosonic waves is also from the statistics of the Van Allen Probes wave data with the wave frequency from proton gyrofrequency to lower hybrid frequency[33]. According to numerous previous studies, the wave normal angle distributions for all these four waves are assumed to be Gaussian[9,10,12,21,23,24,26,27,33,38,39,41-43,45]. The wave parameters of wave

spectral property, wave normal angle distribution, and latitudinal distribution are listed in Supplementary Table 1.

Test particle simulations for coherent plasmaspheric hiss and VLF transmitter waves. In this section, we perform test particle simulations to calculate the electron diffusion coefficients due to coherent hiss and VLF transmitter waves at *L* = 2.3, which is a representative *L*-shell where the bifurcation of energetic electron belt was formed during this event, and compare the results to the quasi-linear diffusion coefficients. We assume that these two types of plasma waves have a single wave frequency (to represent the most coherent wave), which is adopted from the central wave frequency of the statistical wave frequency spectra[12,43]. Recent statistical results demonstrated that plasmaspheric hiss at low *L*-shells mostly undergoes quasi-field-aligned propagation with the wave normal angle mostly less than $30°$[46]. For simplicity, we assume the wave normal angle (θ) of hiss and VLF transmitter waves to be $\theta = 0°$. It is worth noting that the pitch angle scattering effect due to plasmaspheric hiss becomes less efficient with increasing wave normal angle[47-49]. The test particle simulations numerically solve the full electron momentum equation[50-52],

$$\frac{d\boldsymbol{p}}{dt} = q_\mathrm{e}\left(\mathbf{E}_\mathrm{w} + \frac{\boldsymbol{p}}{m_\mathrm{e}\gamma} \times (\mathbf{B}_\mathrm{w} + \mathbf{B}_0)\right), \qquad (1)$$

where $\boldsymbol{p}$ and q_e are the electron momentum and charge, and $\gamma = (1 - v^2/c^2)^{-1/2}$ is the relativistic Lorentz factor where v and *c* are the electron speed and light speed, respectively. $\mathbf{E}_\mathrm{w}$ and $\mathbf{B}_\mathrm{w}$ are the wave electric and magnetic field, and $\mathbf{B}_0$ is the background magnetic field. The momentum equation (Supplementary Equation 1) can be rewritten as a set of three gyro-averaged ordinary differential equations[50-53], which is then numerically solved to perform the test particle simulations. The detailed wave parameters for test particle simulations including wave frequency, wave normal angle, wave latitudinal variations, and wave amplitude are listed in

Supplementary Table 2. Plasma waves are launched from the equator and propagate to higher magnetic latitudes until the maximum magnetic latitude ($\lambda_{\max}$) in the northern hemisphere. Energetic electrons are released at the latitude of the lower value between $\lambda_{\max}$ and the mirror latitude in the northern hemisphere, and move towards the equator. We trace the electrons until they reach the equator for the first time. For each initial energy and pitch angle, 72 electrons are released with their initial phases uniformly distributed between 0° and 360°. Following the diffusion rate calculations in the main text, the hiss wave amplitude is based on the statistical results from the Van Allen Probes measurements[12], and the wave amplitude of VLF transmitter waves is obtained from in situ satellite measurements. For comparison, we also calculate the quasi-linear diffusion coefficients by adopting input parameters similar to those used in the test particle simulations, which are also listed in Supplementary Table 2. Only the first order cyclotron resonance ($N = -1$ for R-mode) is effective in the resonant interactions between energetic electrons and field-aligned propagating whistler mode waves. Although strong dayside hiss can be occasionally observed with the wave amplitude up to a few hundred pT[30], the hiss wave amplitude was observed to be relatively weak, i.e., generally less than tens of pT, during this quiet event.

Supplementary Fig. 4 presents the bounce-averaged pitch-angle diffusion coefficients due to coherent plasmaspheric hiss and VLF transmitter waves calculated using test particle simulations and quasi-linear theory, respectively. Overall, the test particle simulation results agree well with the quasi-linear calculation results, due to the fact that the wave amplitudes of plasmaspheric hiss and VLF transmitter waves are not sufficiently strong (< 25 pT) to cause a significant nonlinear effect. Our results are also consistent with the previous study[53], which indicates that bounce-averaged quasi-linear diffusion coefficients are still valid for narrowband whistler mode waves,

as long as the wave amplitude is small (< a few hundred pT). Therefore, the coherent properties of plasmaspheric hiss or VLF transmitter waves do not significantly affect our quasi-linear simulation results.

Radial Diffusion. Inward radial diffusion driven by ultra-low-frequency waves during geomagnetically disturbed periods is closely associated with fluctuations of magnetospheric convection electric fields, and can cause considerable enhancements of energetic electron flux. However, previous studies have demonstrated that the electron acceleration timescale due to radial diffusion varies from hundreds to thousands of days under quiet geomagnetic conditions at low L-shells[54,55]. To evaluate the effect of radial diffusion for the event under investigation, we follow a commonly adopted empirical model of the radial diffusion coefficients[56], which agrees well with radial diffusion coefficients at the low L-shell region for tens to hundreds of keV electrons estimated based on the Van Allen Probes observations during quiet times[54], given by

$$D_{LL}^{\mathrm{E}} = \frac{1}{4}\left(\frac{cE_{\mathrm{rms}}}{B_0}\right)^2 \left[\frac{T}{1+(\omega_{\mathrm{D}}T/2)^2}\right] L^6, \quad (2)$$

$$D_{LL}^{\mathrm{B}} = 10^{(0.506\mathrm{Kp}-9.325)} L^{10}, \quad (3)$$

$$D_{LL}(\mathrm{Kp}) = D_{LL}^{\mathrm{E}} + D_{LL}^{\mathrm{B}} \text{ (in units of day}^{-1}\text{)}, \quad (4)$$

where B_0 is the dipole magnetic field intensity at the Earth's surface near the magnetic equator (0.311 G), $\omega_{\mathrm{D}} = \left(\frac{3\mu c}{eL^2 R_{\mathrm{E}}^2}\right)\left(1+\frac{2\mu B}{E_0}\right)^{-1/2}$ is the electron drift frequency, μ is the first adiabatic invariant, B is the local magnetic field, e is the unit charge, c is the speed of light, E_0 is the electron rest energy (0.511 MeV), and $E_{\mathrm{rms}}(\mathrm{Kp}) = 0.26(\mathrm{Kp}-1) + 0.1$ mV m^{-1} with Kp = 1 to 6. The total Kp-dependent diffusion coefficient D_{LL} includes both electrostatic D_{LL}^{E} and electromagnetic D_{LL}^{B} contributions. As shown in Supplementary Fig. 7, during periods of quiet geomagnetic activity (Kp = 1 and 2), the radial diffusion coefficients are one or two orders of magnitude smaller than the pitch-angle diffusion rates due to VLF transmitter waves (Fig. 3a-c)

at $L < \sim 2.5$, indicating that radial diffusion plays a minor role in driving the electron dynamics at these low *L*-shells.

Supplementary Discussions

Other Events of the Bifurcated Energetic Electron Belt. While the present study has focused on one specific event to quantitatively unravel the physics accounting for the formation of a bifurcated electron belt at tens of keV, such a bifurcation structure of the energetic electron radial profile is not unique but is occasionally observed by the Van Allen Probes. Supplementary Fig. 10 displays two more events of the bifurcated energetic electron belt observed during the periods of 14–26 October 2016 (left panels) and 10–19 January 2017 (right panels). The bifurcation structure lasted for ~10 days in October 2016 and ~8 days in January 2017, both corresponding to the relatively quiet geomagnetic conditions (Supplementary Fig. 10a, b, g, h).

Because ground-based VLF transmitters are generally operated continuously with nearly constant power except during rare periods of maintenance, their modulation of the natural energetic electron environment is expected to take place continuously on typical timescales of ~ 10 days due to the relatively weak intensity of VLF transmitter waves in space. Both observational (Fig. 1, Supplementary Figs. 1 &10) and theoretical (Fig. 3 & Supplementary Fig. 8) results have indicated that the bifurcation of the near-Earth electron belt was formed gradually on the similar timescales and became pronounced with a long duration. However, in order for the effect of VLF transmitters to become discernible in space, the disturbance level of geomagnetic activity needs to be relatively low (*e.g.,* $Kp < 3$) for at least several days so that the resultant bifurcation structure of energetic electron fluxes develops evidently to become detectable. Otherwise, naturally driven electron dynamics in association with enhanced geomagnetic

activities can either remove the bifurcation structure by electron refilling or exceed the contribution of VLF transmitters.

Supplementary Figures

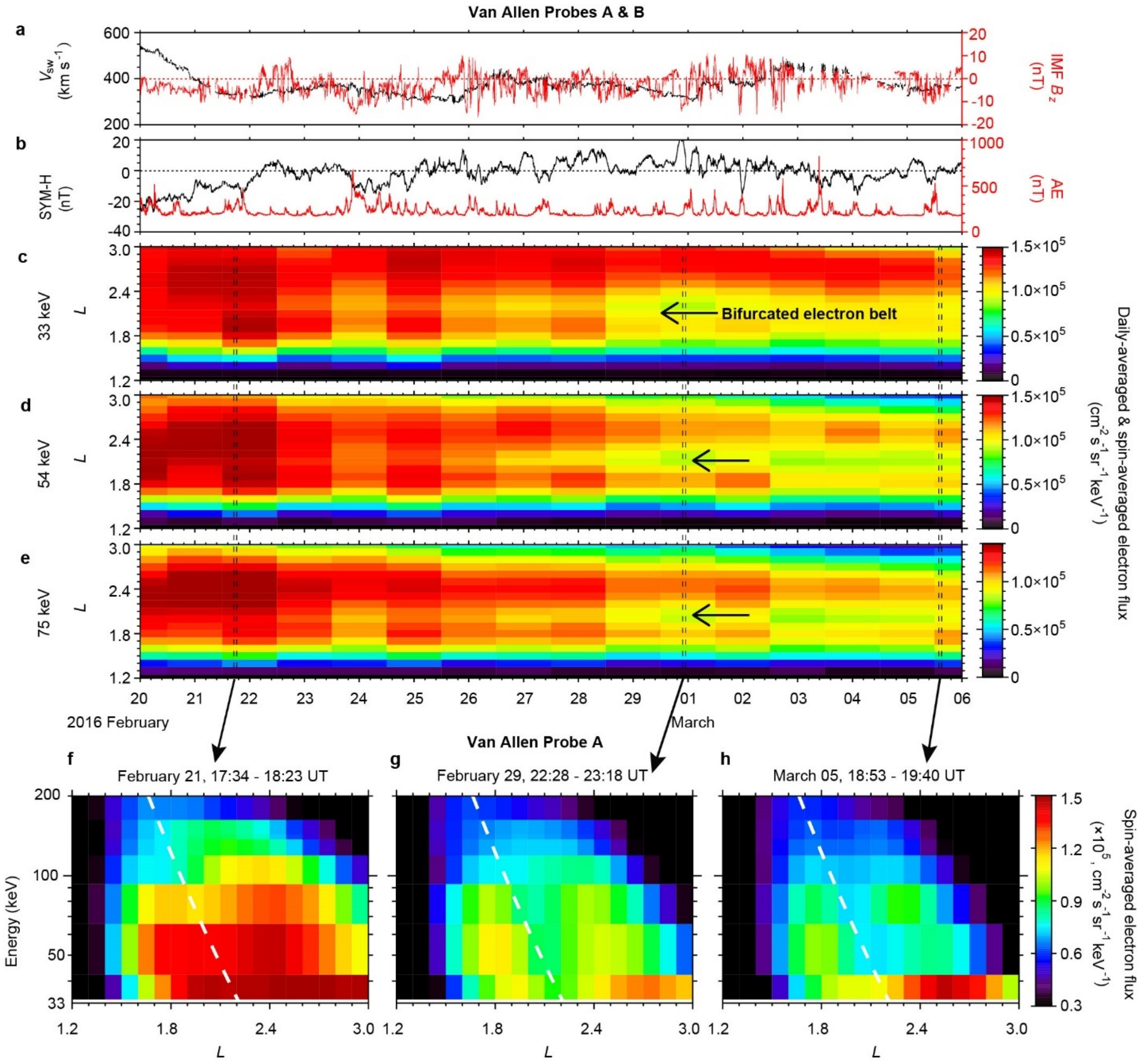

Supplementary Figure 1. Radial profiles of energetic electron fluxes from 20 February to 06 March 2016. The same format as Fig. 1, except using the measurements from MagEIS with fewer energy channels below 200 keV[3]. The white dashed curves indicate the minimum first-order cyclotron resonant energies of electrons interacting with 24 kHz VLF transmitter waves at the geomagnetic equator.

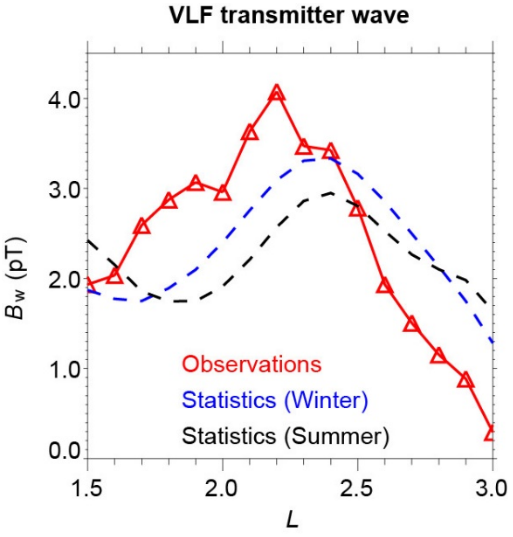

Supplementary Figure 2. Comparison of in situ observed wave amplitude of VLF transmitter waves to the statistical values[43]. The radial profile of the root-mean-square (RMS) magnetic wave amplitudes of VLF transmitter waves averaged over all MLTs and the entire 15-day period (same as the red triangle curve in Fig. 2c), and the statistical RMS wave amplitude of VLF transmitter waves during the northern hemispheric winter (blue) and summer[43] (black).

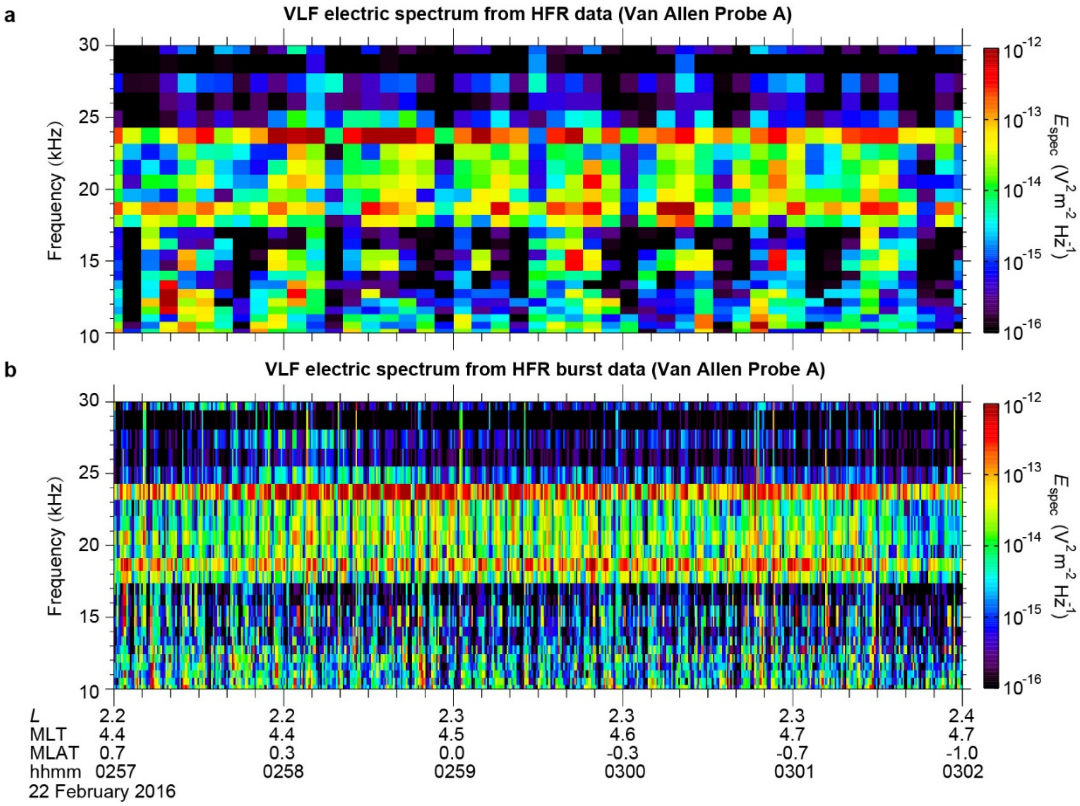

Supplementary Figure 3. **In situ observations of coherent VLF transmitter waves. a**, An example of electric power spectrogram from the survey-mode HFR, and **b**, burst-mode HFR measurements by Van Allen Probe A on 22 February 2016. The corresponding *L*-shell, magnetic local time (MLT), and magnetic latitude (MLAT) along the satellite trajectory are also labeled accordingly.

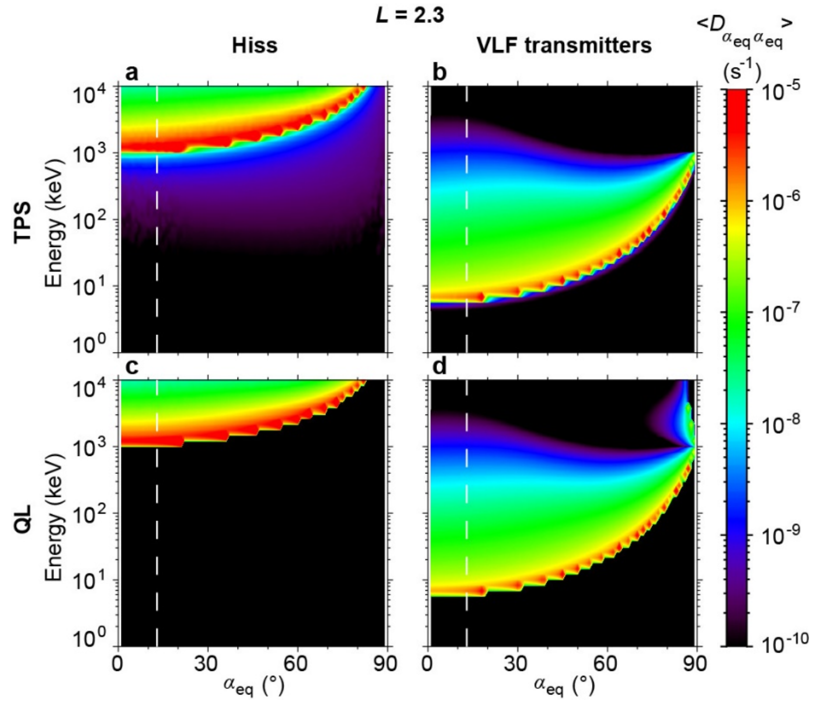

Supplementary Figure 4. Comparisons of the pitch-angle diffusion coefficients due to coherent plasmaspheric hiss and VLF transmitter waves calculated using test particle simulations and quasi-linear theory. Bounce-averaged electron pitch-angle diffusion coefficients (<$D_{\alpha_{\mathrm{eq}}\alpha_{\mathrm{eq}}}$>) as a function of equatorial pitch-angle (α_{eq}) and electron energy at *L* = 2.3 for coherent plasmaspheric hiss (left) and VLF transmitter waves (right) calculated using test particle simulations (a-b) and the corresponding quasi-linear calculation results (c-d). The vertical white lines represent the equatorial bounce loss cone at *L* = 2.3. The detailed wave parameters used for the calculations are listed in Supplementary Table 2.

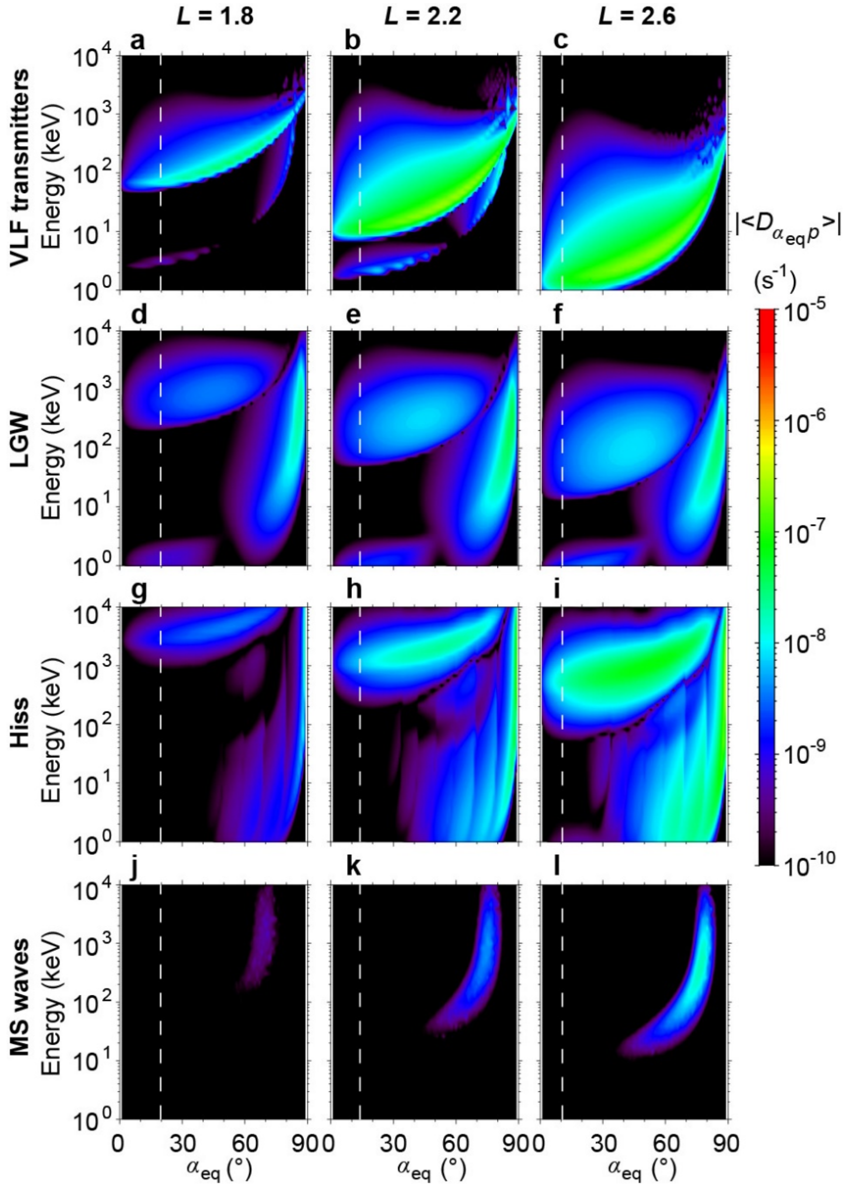

Supplementary Figure 5. Computation of drift- and bounce-averaged electron cross diffusion coefficients ($|\langle D_{\alpha_{eq}p} \rangle|$) due to VLF transmitter waves and naturally occurring plasma waves at L = 1.8, 2.2, and 2.6. The format is the same as Fig. 3a-l. The vertical white dashed lines in **a-l** represent the equatorial bounce loss cone at the given L-shell.

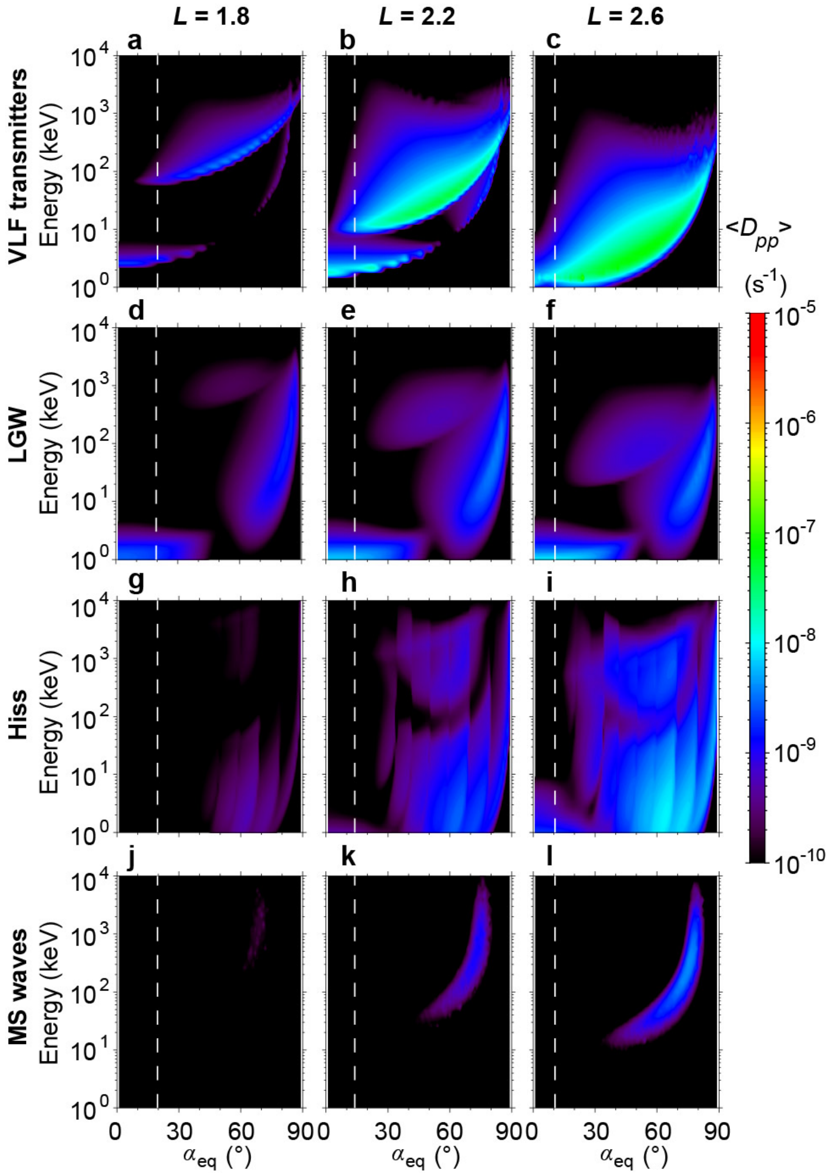

Supplementary Figure 6. Computation of drift- and bounce-averaged electron momentum diffusion coefficients (<D_{pp}>) due to VLF transmitter waves and naturally occurring plasma waves at *L* = 1.8, 2.2, and 2.6. The format is the same as Fig. 3a-l. The vertical white dashed lines in **a-l** represent the equatorial bounce loss cone at the given *L*-shell.

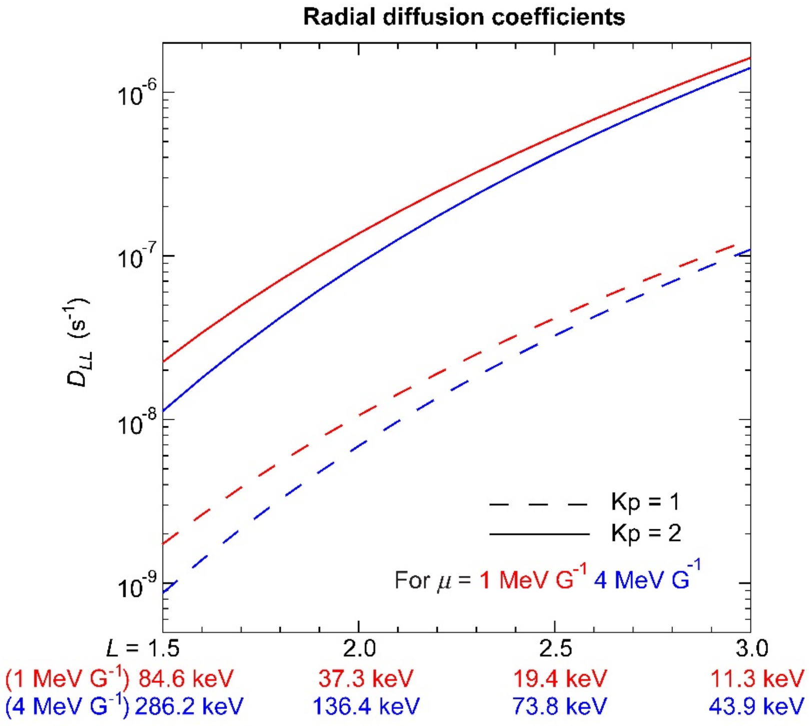

Supplementary Figure 7. Radial diffusion coefficients under geomagnetically quiet conditions[56]. The results are calculated for the two representative values of the first adiabatic invariant, *i.e.,* μ = 1 MeV G^{-1} (red curves) and 4 MeV G^{-1} (blue curves) and two typical values of Kp index, *i.e.,* Kp = 1 (dashed curves) and Kp = 2 (solid curves). The electron kinetic energies marked at the bottom are calculated for the selected μ at 90° pitch angle at the corresponding L-shell.

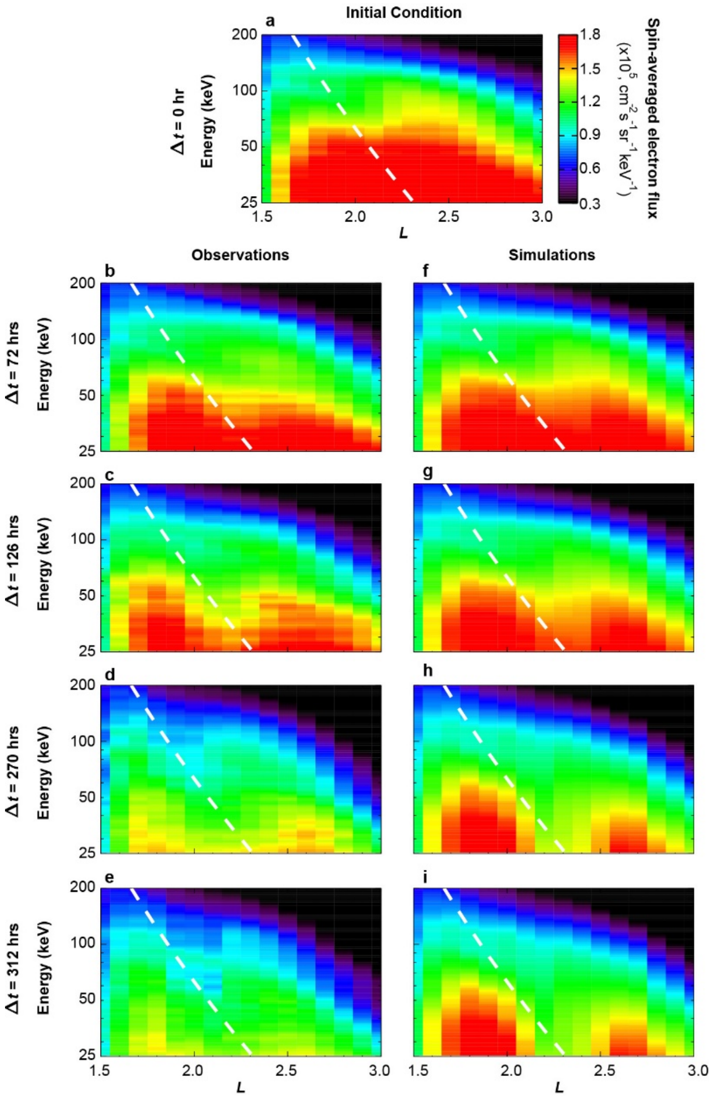

Supplementary Figure 8. Comparison of electron energy spectra between simulations and observations at *L* ~ 1.5–3.0. a, The initial conditions of electron fluxes collected by RBSPICE during 15–21 UT on 21 February when the satellites were near the equator (|MLAT| < 5°). **b-e**, Radial profiles of spin-averaged electron flux from the RBSPICE observations at the time stamps of 72, 126, 270 and 312 hours after 18 UT on 21 February 2016, displayed in the same format as that in Fig. 1f-h. **f-i**, Same format as b-e but from the Fokker-Planck diffusion simulations. The white dashed lines indicate the minimum first-order cyclotron resonant energies of electrons interacting with 24 kHz VLF transmitter waves at the geomagnetic equator.

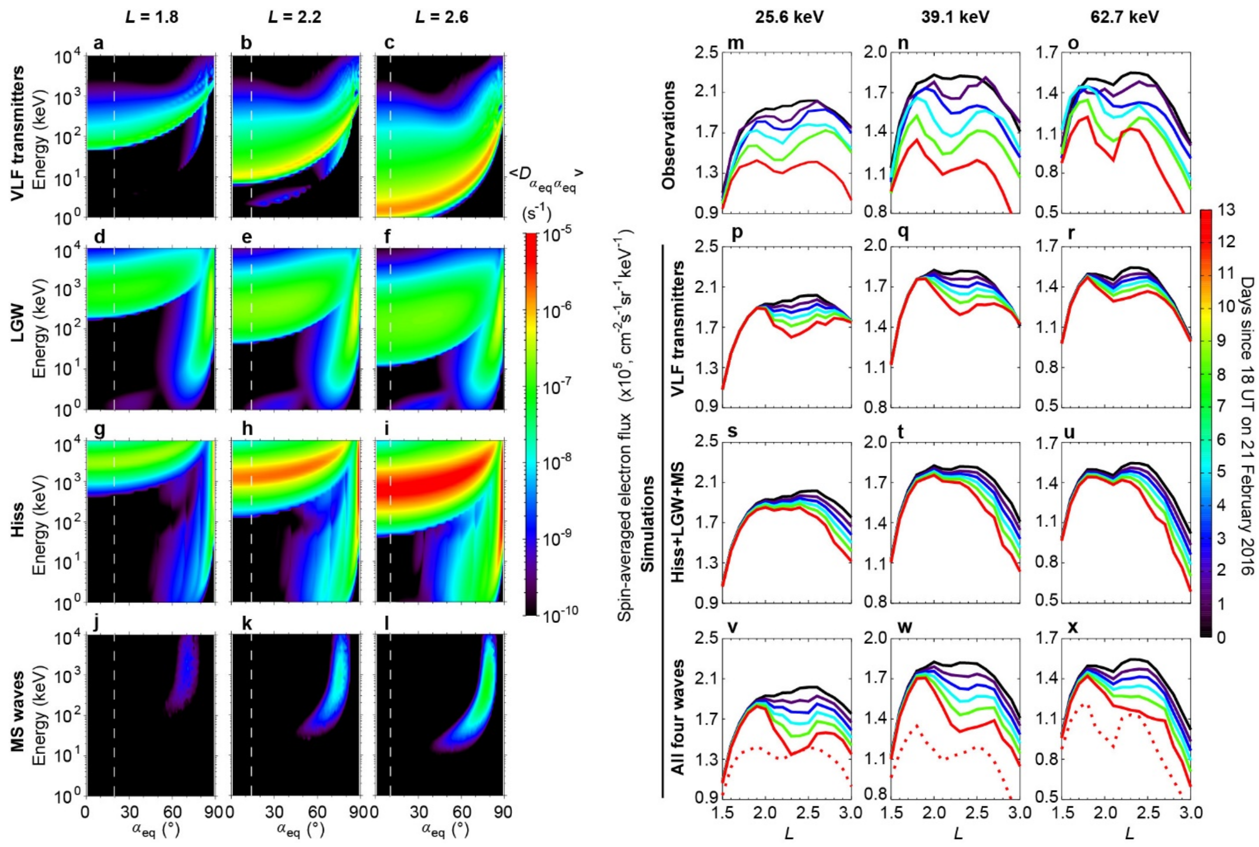

Supplementary Figure 9. Computation of electron pitch-angle diffusion coefficients due to individual wave modes (a-l) and comparisons of Fokker-Planck simulation results to observations (m-x). The format is the same as Fig. 3, except for using the statistical wave amplitudes of VLF transmitter waves during the northern hemisphere winter.

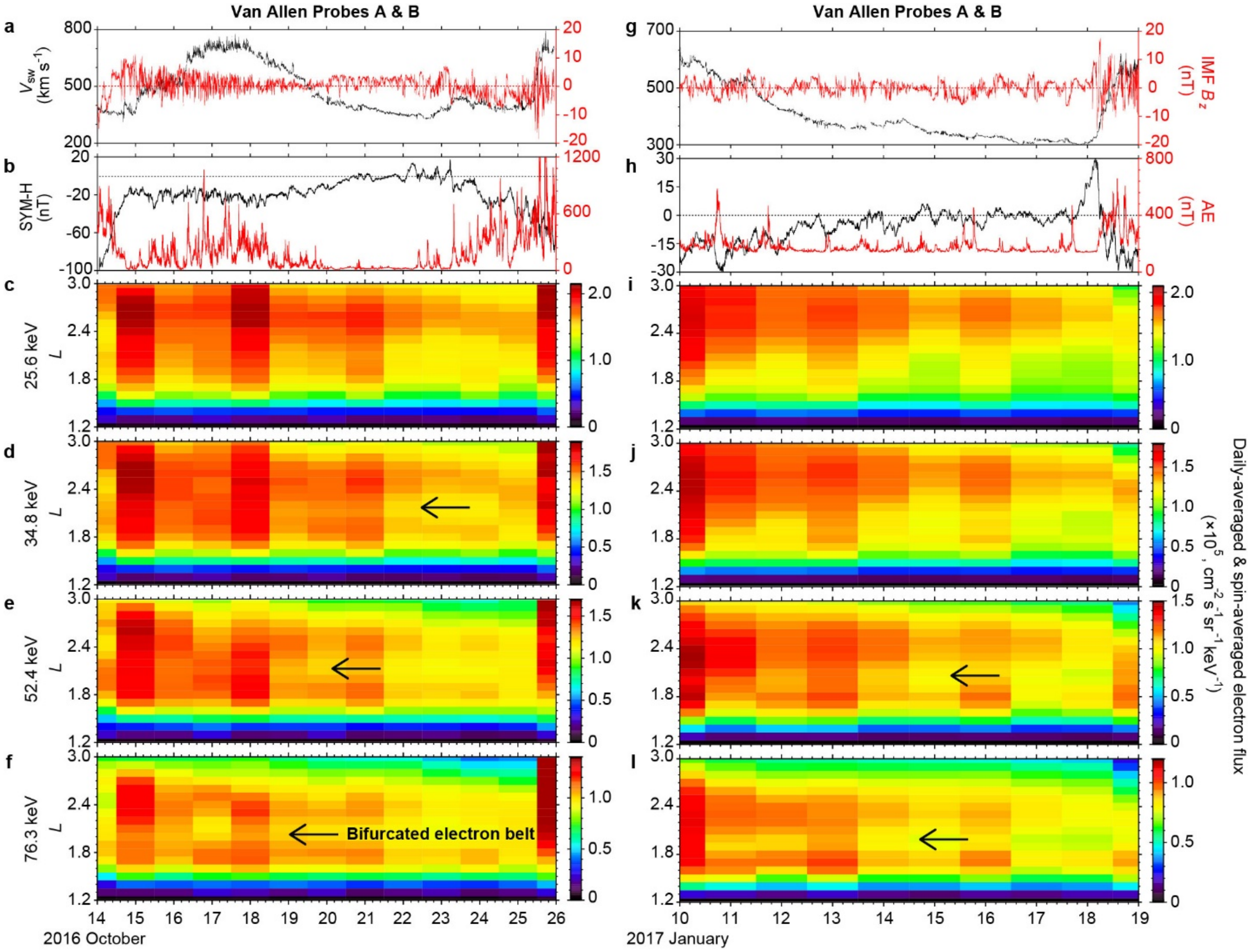

Supplementary Figure 10. RBSPICE observations of the bifurcation structure of near-Earth energetic electron belt during two other specific periods. a-f, The event of 14–26 October 2016. **g-l**, The event of 10–19 January 2017. For both events, the format is the same as that in Fig. 1a-e.

Supplementary Tables

Supplementary Table 1. Input wave parameters adopted for calculations of electron diffusion coefficients over L ~ 1.5–3.0.

Wave types	Wave spectrum	Amplitude	WNA distribution†	MLAT coverage	Resonance harmonics
VLF transmitter waves	Ma et al.[43]	Satellite measurements during the observed event (red curve in Fig. 2c)	$L \leq 1.7$: Gaussian with $\theta_{min} = 10^o$, $\theta_{max} = 80^o$, $\theta_m = 50^o$, $\theta_w = 10^o$ $L > 1.7$: Gaussian with $\theta_{min} = 0^o$, $\theta_{max} = 30^o$, $\theta_m = 0^o$, $\theta_w = 10^o$	|MLAT| ≤ latitude of magnetic field line at the altitude of 800 km	Landau and cyclotron resonances with up to 10 orders of resonant harmonics
Lightning-generated whistlers	Green et al.[44]	Green et al.[44] (black curve in Fig. 2d)	Gaussian with $\theta_{min} = 0^o$, $\theta_{max} = 70^o$, $\theta_m = 0^o$, $\theta_w = 40^o$	|MLAT| ≤ latitude of magnetic field line at the altitude of 800 km	Landau and cyclotron resonances with up to 10 orders of resonant harmonics
Plasmaspheric hiss	Li et al.[12]	Li et al.[12] (AL dependent; green curve in Fig. 2d)	$L \leq 1.7$: Gaussian with $\theta_{min} = 0^o$, $\theta_{max} = 45^o$, $\theta_m = 20^o$, $\theta_w = 30^o$ $L > 1.7$: Latitudinally varying model of Ni et al.[23]	$L \leq 2.2$: |MLAT| ≤latitude of magnetic field line at the altitude of 800 km $L > 2.2$: |MLAT| ≤ 45°	Landau and cyclotron resonances with up to 10 orders of resonant harmonics
Magnetosonic waves	Ma et al.[33]	Ma et al.[33] (AE dependent; magenta curve in Fig. 2d)	Gaussian with $\theta_{min} = 88^o$, $\theta_{max} = 89.3^o$, $\theta_m = 89^o$, $\theta_w = 86^o$	|MLAT| ≤ 3°	Landau resonance

† Wave power is proportional to exp $\left(-\frac{(\tan\theta - \tan\theta_m)^2}{\tan^2\theta_w}\right)$, and $\theta_{min} \leq \theta \leq \theta_{max}$.

Supplementary Table 2. Input wave parameters adopted for test particle simulations (TPS) and quasi-linear (QL) calculations at L = 2.3.

Wave type	Method	Wave spectrum†	WNA distribution‡	Amplitude	MLAT coverage	Resonance harmonics
Plasmaspheric hiss	TPS	252 Hz	0°	24.31 pT	$\lvert MLAT \rvert \leq 45°$	Not applicable
Plasmaspheric hiss	QL	Gaussian with f_m = 252 Hz, f_{min} = 251 Hz, f_{max} =253 Hz, f_w = 2 Hz.	Gaussian with θ_{min} = 0°, θ_{max} = 3°, θ_m = 0°, θ_w = 3°	24.31 pT	$\lvert MLAT \rvert \leq 45°$	First order cyclotron resonance
VLF transmitter waves	TPS	24 kHz	0°	3.47 pT	$\lvert MLAT \rvert \leq 45.6°$	Not applicable
VLF transmitter waves	QL	Gaussian with f_m = 24 kHz, f_{min} = 23.9 kHz, f_{max} =24.1 kHz, f_w = 0.1 kHz.	Gaussian with θ_{min} = 0°, θ_{max} = 3 °, θ_m = 0°, θ_w = 3°	3.47 pT	$\lvert MLAT \rvert \leq 45.6°$	First order cyclotron resonance

---

† For Gaussian wave frequency (f) distribution, wave power is proportional to exp $(-\frac{(f-f_m)^2}{{f_w}^2})$, and $f_{min} \leq f \leq f_{max}$.

‡ For Gaussian wave normal angle (θ) distribution, wave power is proportional to exp $(-\frac{(\tan\theta-\tan\theta_m)^2}{\tan^2\theta_w})$, and $\theta_{min} \leq \theta \leq \theta_{max}$.

Supplementary References

1. Mauk, B. H. et al. Science objectives and rationale for the Radiation Belt Storm Probes mission. *Space Sci. Rev.* **179**, 3-27 (2013).
2. Mitchell, D. G. et al. Radiation belt storm probes ion composition experiment (RBSPICE). *The Van Allen Probes Mission*, 263-308. (Springer, Boston, MA, 2013).
3. Blake, J. B. et al. The Magnetic Electron Ion Spectrometer (MagEIS) instruments aboard the Radiation Belt Storm Probes (RBSP) spacecraft. *Space Sci. Rev.* **179**, 383-421 (2013).
4. Spence, H. E. et al. Science goals and overview of the Energetic Particle, Composition, and Thermal Plasma (ECT) Suite on NASA's Radiation Belt Storm Probes (RBSP) Mission. *Space Sci. Rev.* **179**, 311-336 (2013).
5. Baker, D. N. et al. The relativistic electron-proton telescope (REPT) instrument on board the Radiation Belt Storm Probes (RBSP) spacecraft: Characterization of Earth's radiation belt high-energy particle populations. *Space Sci. Rev.* **179**, 337-381 (2013).
6. Kletzing, C. A. et al. The Electric and Magnetic Field Instrument Suite and Integrated Science (EMFISIS) on RBSP. *Space Sci. Rev.* **179**, 127181 (2013).
7. Cerisier, J. C. A theoretical and experimental study of non-ducted VLF waves after propagation through the magnetosphere. *J. Atmos. Terr. Phys.* **35**, 77-94 (1973).
8. Clilverd, M. A. et al. Ground-based transmitter signals observed from space: Ducted or nonducted?. *J. Geophys. Res.* **113**, A04211 (2008).
9. Abel, B. & Thorne, R. M. Electron scattering loss in Earth's inner magnetosphere: 2. Sensitivity to model parameters. *J. Geophys. Res. Space Phys*. **103**, 2397-2407 (1998).
10. Claudepierre, S. G. et al. Empirically estimated electron lifetimes in the Earth's radiation belts: Comparison with theory. *Geophys. Res. Lett.* **47**. (2020).

11. Thorne, R. M. Radiation belt dynamics: The importance of wave-particle interactions. *Geophys. Res. Lett.* **37**, L22107 (2010).

12. Li, W. et al. Statistical properties of plasmaspheric hiss derived from Van Allen Probes data and their effects on radiation belt electron dynamics. *J. Geophys. Res. Space Phys.* **120**, 3393-3405 (2015).

13. Tsurutani, B. T., Falkowski, B. J., Pickett, J. S., Santolik, O. & Lakhina, G. S. Plasmaspheric hiss properties: Observations from Polar. *J. Geophys. Res. Space Phys*. **120**, 414–431(2015).

14. Thorne, R. M., Smith, E. J., Burton, R. K. & Holzer, R.E. Plasmaspheric hiss. *J. Geophys. Res.* **78**, 1581 (1973).

15. Meredith, N. P., Horne, R. B., Thorne, R. M., Summers, D. & Anderson, R. R. Substorm dependence of plasmaspheric hiss. *J. Geophys. Res.* **109**, 6209 (2004).

16. Li, W. et al. An unusual enhancement of low-frequency plasmaspheric hiss in the outer plasmasphere associated with substorm injected electrons. *Geophys. Res. Lett.* **40**, 3798–3803 (2013).

17. Shi, R. et al. Properties of whistler mode waves in Earth's plasmasphere and plumes. *J. Geophys. Res. Space Phys.* **124**, 1035– 1051 (2019).

18. Zhang, W. et al. Statistical properties of hiss in plasmaspheric plumes and associated scattering losses of radiation belt electrons *Geophys. Res. Lett.* **46**, 5670-5680 (2019).

19. Ma, Q. et al. Characteristic energy range of electron scattering due to plasmaspheric hiss, *J. Geophys. Res. Space Phys*. **121**, 11,737– 11,749 (2016).

20. Tsurutani, B. T., Smith, E. J. & Thorne, R. M. Electromagnetic hiss and relativistic electron losses in the inner zone. *J. Geophys. Res.* **80**, 600-607 (1975).

21. Meredith, N. P., Horne, R. B., Glauert, S. A. & Anderson, R. R. Slot region electron loss

timescales due to plasmaspheric hiss and lightning-generated whistlers. *J. Geophys. Res.* **112**, A08214 (2007).

22. Summers, D. et al. Electron scattering by whistler-mode ELF hiss in plasmaspheric plumes. *J. Geophys. Res.* **113**, A04219 (2008).
23. Ni, B., Bortnik, J., Thorne, R. M., Ma, Q. & Chen, L. Resonant scattering and resultant pitch angle evolution of relativistic electrons by plasmaspheric hiss, *J. Geophys. Res. Space Phys.* **118**, 7740-7751 (2013).
24. Ni, B. et al. Resonant scattering of energetic electrons by unusual low-frequency hiss. *Geophys. Res. Lett.* **41**, 1854-1861 (2014).
25. Lyons, L. R. & Thorne, R. M. Equilibrium structure of radiation belt electrons. *J. Geophys. Res.* **78**, 2142 (1973).
26. Zhao, H. et al. Plasmaspheric hiss waves generate a reversed energy spectrum of radiation belt electrons. *Nature Phys.* **15**, 367-372 (2019).
27. Ni, B. et al. Parametric sensitivity of the formation of reversed electron energy spectrum caused by plasmaspheric hiss. *Geophys. Res. Lett.* **46**, 4134-4143 (2019).
28. Tsurutani, B. T. et al. Plasmaspheric hiss: Coherent and intense. *J. Geophys. Res. Space Phys*. **123**, 10009S10029 (2018).
29. Tsurutani, B. T. et al. Low frequency (f < 200 Hz) polar plasmaspheric hiss: coherent and intense. *J. Geophys. Res. Space Phys*. **124**, 10063– 10084 (2019).
30. Falkowski, B. J., Tsurutani, B. T., Lakhina, G. S. & Pickett, J. S. Two sources of dayside intense, quasi-coherent plasmaspheric hiss: A new mechanism for the slot region?. *J. Geophys. Res. Space Phys*. **122**,1643–1657 (2017).
31. Bortnik, J., Inan, U. S. & Bell, T. F. Frequency-time spectra of magnetospherically reflecting

whistlers in the plasmasphere. *J. Geophys. Res.* **108**, 1030 (2003).

32. Voss, H. D., Walt, M., Imhof, W. L., Mobilia, J. & Inan, U. S. Satellite observations of lightning-induced electron precipitation. *J. Geophys. Res.* **103**, 11725 (1998).
33. Ma, Q. et al. Electron scattering by magnetosonic waves in the inner magnetosphere. *J. Geophys. Res. Space Phys.* **121**, 274-285 (2016).
34. Russell, C. T. & Holzer, R. E. OGO 3 observation of ELF noise in the magnetosphere 2. The nature of the equatorial noise. *J. Geophys. Res. 75*, 755–768 (1970).
35. Santolik, O., Pickett, J. S. & Gurnett, D. A. Spatiotemporal variability and propagation of equatorial noise observed by Cluster. *J. Geophys. Res.* **107**, 1495 (2002).
36. Santolk, O., et al. Systematic analysis of equatorial noise below the lower hybrid frequency. *Ann. Geophys.* **22**, 2587–2595 (2004).
37. Ma, Q. et al. Global survey and empirical model of fast magnetosonic waves over their full frequency range in earth's inner magnetosphere. *J. Geophys. Res. Space Phys.* **124**, 10270–10282 (2019).
38. Horne, R. B. et al. Electron acceleration in the Van Allen radiation belts by fast magnetosonic waves. *Geophy. Res. Lett.* **3**4, S09S03 (2007).
39. Xiao, F. et al., Wave-driven butterfly distribution of Van Allen belt relativistic electrons. *Nature Comm.* **6**, 8590 (2015).
40. Shprits, Y. Y. Estimation of bounce resonant scattering by fast magnetosonic waves. *Geophys. Res. Lett.* **43**, 998–1006 (2016).
41. Li, J. et al. Formation of energetic electron butterfly distributions by magnetosonic waves via Landau resonance. *Geophys. Res. Lett.* **43**, 3009–3016 (2016).

42. Fu, S., Ni, B., Zhou, R., Cao, X. & Gu X. Combined scattering of radiation belt electrons caused by Landau and bounce resonant interactions with magnetosonic waves. *Geophys. Res. Lett.* **46**, 10313-10321 (2019).

43. Ma, Q., Mourenas, D., Li, W., Artemyev, A. & Thorne, R. M. VLF transmitters from ground-based transmitters observed by the Van Allen Probes: Statistical model and effects on plasmaspheric electrons. *Geophys. Res. Lett.* **44**, 6483-6491 (2017).

44. Green et al. Properties of Lightning Generated Whistlers Based on Van Allen Probes Observations and Their Global Effects on Radiation Belt Electron Loss. *Geophys. Res. Lett.* **47,** e2020GL089584 (2020).

45. Ross, J. P., Meredith, N. P., Glauert, S. A., Horne, R. B. & Clilverd, M. A. Effects of VLF transmitter waves on the inner belt and slot region. *J. Geophys. Res. Space Phys.* **124**, 5260-5277 (2019).

46. Hartley, D. P., Kletzing, C. A., Santolík, O., Chen, L., & Horne, R. B. Statistical properties of plasmaspheric hiss from Van Allen Probes observations. *J. Geophys. Res. Space Phys.* **123**, 2605– 2619 (2018).

47. Meredith, N. P. et al. Energetic outer zone electron loss timescales during low geomagnetic activity, *J. Geophys. Res.* **111**, A05212 (2006).

48. Ripoll, J.-F., Albert, J. M., & Cunningham, G. S. Electron lifetimes from narrowband wave-particle interactions within the plasmasphere, *J. Geophys. Res. Space Phys.* **119**, 8858– 8880 (2014).

49. Gao, Y., et al. Influence of wave normal angles on hiss-electron interaction in Earth's slot region, *J. Geophys. Res. Space Phys.* **120**, 9385– 9400 (2015).

50. Bell, T. F. The nonlinear gyroresonance interaction between energetic electrons and coherent

VLF waves propagating at an arbitrary angle with respect to the Earth's magnetic field. J. Geophys. Res. 89, 905–918 (1984).

51. Bortnik, J., Thorne, R. M., & Inan, U. S. Nonlinear interaction of energetic electrons with large amplitude chorus. *Geophys. Res. Lett.* **35**, L21102 (2008).

52. Li, J. et al. Comparison of formulas for resonant interactions between energetic electrons and oblique whistler-mode waves. *Physics of Plasmas*. **22**, 052902 (2015).

53. Tao, X., Bortnik, J., Albert, J. M. & Thorne, R. M. Comparison of bounce-averaged quasi-linear diffusion coefficients for parallel propagating whistler mode waves with test particle simulations, *J. Geophys. Res.* **117**, A10205 (2012).

54. O'Brien, T. P. et al. Inner zone and slot electron radial diffusion revisited. *Geophys. Res. Lett.* **43**, 7301–7310 (2016).

55. Zhao, H. & Li, X. Modeling energetic electron penetration into the slot region and inner radiation belt. *J. Geophys. Res. Space Phys.* **118**, 6936–6945 (2013).

56. Brautigam, D. H. & Albert, J. M. Radial diffusion analysis of outer radiation belt electrons during the October 9, 1990, magnetic storm. *J. Geophys. Res.* **105**, 291–309 (2000).
